# The Sister Chromatid Division of the Heteromorphic Sex Chromosomes in *Silene* Species and Their Transmissibility towards the Mitosis

**DOI:** 10.3390/ijms23052422

**Published:** 2022-02-22

**Authors:** Václav Bačovský, Tomáš Janíček, Roman Hobza

**Affiliations:** Department of Plant Developmental Genetics, Institute of Biophysics of the Czech Academy of Sciences, Kralovopolska 135, 612 00 Brno, Czech Republic; xbacovs@ibp.cz (V.B.); tjanicek@ibp.cz (T.J.)

**Keywords:** sex chromosomes, central interpolar axis, sister chromatid division, chromosome velocity, *Silene*

## Abstract

Young sex chromosomes possess unique and ongoing dynamics that allow us to understand processes that have an impact on their evolution and divergence. The genus *Silene* includes species with evolutionarily young sex chromosomes, and two species of section *Melandrium*, namely *Silene latifolia* (24, XY) and *Silene dioica* (24, XY), are well-established models of sex chromosome evolution, Y chromosome degeneration, and sex determination. In both species, the X and Y chromosomes are strongly heteromorphic and differ in the genomic composition compared to the autosomes. It is generally accepted that for proper cell division, the longest chromosomal arm must not exceed half of the average length of the spindle axis at telophase. Yet, it is not clear what are the dynamics between males and females during mitosis and how the cell compensates for the presence of the large Y chromosome in one sex. Using hydroxyurea cell synchronization and 2D/3D microscopy, we determined the position of the sex chromosomes during the mitotic cell cycle and determined the upper limit for the expansion of sex chromosome non-recombining region. Using 3D specimen preparations, we found that the velocity of the large chromosomes is compensated by the distant positioning from the central interpolar axis, confirming previous mathematical modulations.

## 1. Introduction

Generally accepted theory predicts that the sex chromosomes originate from a single pair of autosomes [1]. Compared to the animals, plant sex chromosomes evolved relatively recently and independently across a wide diversity of taxa, displaying a huge variety of X(Z) and Y(W) chromosome divergence [2]. Despite their independent origin, they share similar evolutionary characteristics, including recombination suppression, Y (W) chromosome degeneration, and dosage compensation, representing exceptional features of genomic convergence [3]. It is assumed that at least two mutations located at different loci or on a different chromosome pair are needed to differentiate the proto-sex chromosomes. As an alternative model, a single master-switch gene may act as the main control element to trigger male or female development [4]. The sex-specific region (one or multiple genes) becomes linked to the sex-determining region which has lower levels of recombination. Reduced recombination occurs due to the selection which favours the linkage between sex-determining and sexually antagonistic genes, allowing a non-recombining genomic landscape to occur [5]. The recombination suppression is however not specific to a concrete region and the new sex-determining genes may arise in the existing recombination cold spot region, as documented in *Neurosposra tetrasperma* [6] or dioecious plant *Carica papaya* [7] and closely related *Vasconcellea parviflora* [8]. Once the proto-sex chromosomes are established, they evolve into fully differentiated sex chromosomes with large non-recombining regions. The successive steps of recombination suppression leads to different level of divergence between the sex chromosomes, allowing the formation of evolutionary strata (stratum = a region with a similar level of synonymous substitution between X- and Y-linked alleles) [9]. The reduced recombination also causes extensive accumulation of deleterious and loss-of-function mutations compared to the X-linked alleles [10]. Overall, the restricted recombination among other processes creates a landscape that has a lower capability to regulate the accumulation of transposable elements and the expansion of various satellite repeats, as documented for the satellitome of the large Y chromosome in *Eneoptera surinamensis* [11] or degenerated Y_1_Y_2_ chromosomes in *Rumex acetosa* [12]. In fact, multiple insertions are assumed as the most important factors that increase the size of the non-recombining region, leading to sex chromosome heteromorphism (different in size). Thus, the TEs accumulation plays a major role in the sex chromosome genomic upsizing, promoting genetic degeneration and chromosomal rearrangements, increasing the level of recombination suppression [13]. The repetitive fraction in the non-recombining region affects local gene expression and may increase the overall methylation of neighbouring sequences, stimulating the process of Y chromosome inactivation. Such sex chromosome upsizing was well was described for the large Y chromosome in *Coccinia grandis* [14] or *Silene* species of section *Melandrium* [13,15]. Therefore, the models with evolutionary young sex chromosomes are important to study the early steps of sex chromosome evolution and answer fundamental questions of sex chromosome biology [5,16].

The section *Melandrium* of the genus *Silene* includes several important species which are models of sex chromosome evolution, namely *S. latifolia* and *S. dioca*. The sex chromosomes in *S. latifolia* evolved 11 MYA [17,18] and originate at least from three pairs of autosomes [15]. The Y chromosome is 1.4 times larger compared to the X chromosome (in *S. dioica* the Y is 1.5 larger), and both sex chromosomes accumulated a large number of TEs and tandem repeats [13,19,20]. The pseudoautosomal region (PAR) is strongly reduced, forming only 10% of the Y chromosome length [15,21]. A large number of Y-linked genes is degenerated or down-regulated, showing moderate gene loss and Y chromosome degeneration [22,23,24]. Y chromosome degeneration is further supported by the low abundance of active histone posttranslational modifications in the Y non-recombining region [25,26]. Despite the ongoing process of degeneration, the sex chromosome size is still very dynamic, based on genomic analysis of several *S. latofolia* ecotypes [20]. Yet, it is unknown what is the upper limit size of the non-recombining region expansion in *Silene* species.

It has been shown that for proper cell division, the longest chromosomal arm in the genome cannot exceed half of the average length of the spindle axis at telophase. The sister chromatid division of such chromosomes will otherwise lead to incomplete mitotic division and chromosome disruption, defining the so-called “upper limit of a chromosome size” [27]. As demonstrated in barley, such incomplete division of disrupted chromatid(s) resulted in a micronuclei formation and impaired fertility and viability of the carrier individuals [28]. Compared to the large chromatids, the chromosomes of smaller than average size do not segregate correctly either [29]. Taken together, variation in chromosome length may impair the fitness of an organism and incorrect cell division leads to micronuclei formation or to cell death [30]. The upper limit of a chromosomal size restricts overall chromosomal size variation. This is because the limit of the cell dimension and spindle extension do not favour chromosomes with significantly different lengths, which in turn decreases evolutionary fitness [31]. Chromosomes of various lengths may also differ in chromosome condensation [32,33], affecting chromosome stiffness and sister chromatid division during mitosis [34]. The decrease in chromosome velocity (movement) was shown to be reduced by specific chromosome positioning so far in *Melanoplus* [35]. Such evidence raises another important question: does the position of the sex chromosomes differ during mitosis and during the transition from metaphase to telophase?

To answer such questions, we studied the mitotic cell division in two dioecious plants, *S. latifolia* and *S. dioica*. We measured the spindle axis extension in telophase and examined the position of the sex chromosome during the transition from metaphase to telophase using hydroxyurea cell synchronization and 2D/3D microscopy preparations. We estimate the upper limit of the sex chromosome size and discuss the X-Y-chromosome arm positioning during sister chromatid division in the context of the chromosome velocity during the mitotic division.

## 2. Results

We analysed the position of the large sex chromosomes in metaphase and their sister chromatid division during anaphase A, anaphase B, and telophase in two dioecious species: *S. latifolia* and *S. dioica*. Sex chromosomes in both species were identified according to their size (both are larger compared to autosomes) and DNA probe combination, namely subtelomeric satellite X43.1 and centromeric repeat STAR-C (see Materials and Methods).

### 2.1. Sister Chromatid Division of the Sex Chromosomes in Dioecious Plants

**Figure 1:**
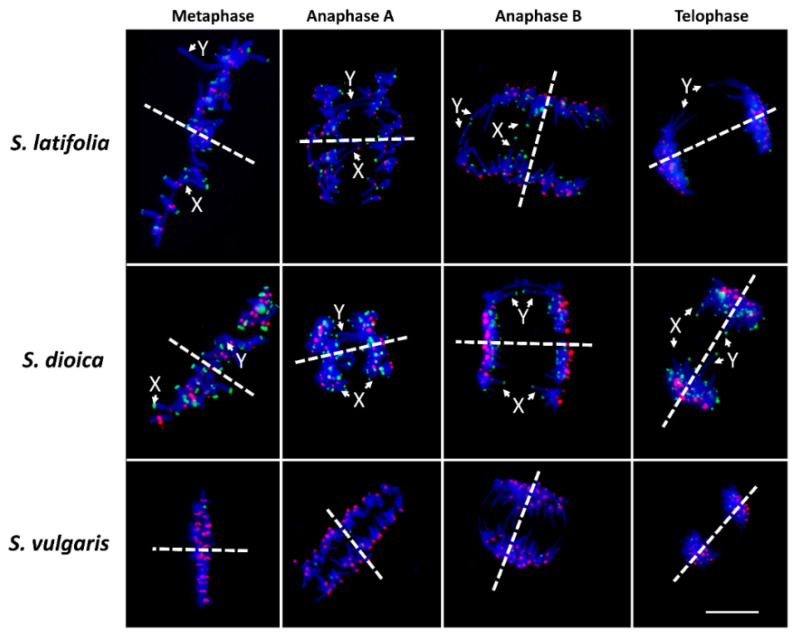
Mitotic cell division in male plants of *S. latifolia* and *S. dioica*, and gynodioecious *S. vulgaris*. During transition from anaphase A to telophase, X and Y chromatids are located towards the spindle equator and distantly from central interpolar axis (dash lines). Subtelomeric satellite X43.1 (green) and centromeric repeat STAR-C (red) were used to distinguish centromeres in *S. latifolia*, *S. dioica* and *S. vulgaris*, and sex chromosomes in *S. latifolia* and *S. dioica*. Chromosomes were counterstained with DAPI (blue). Scale bar = 10 µm.

The sex chromosomes in metaphase were distantly located from the central interpolar axis in both studied dioecious species. Such distant localization is clearly visible through the progression from metaphase to anaphase B (Figure 1). Simultaneously, the Y chromosome chromatids are still attached in Y q-arm (X43.1 enriched region) during anaphase A and continue to protrude towards the division plane in anaphase B and in telophase (Figure 1 and Figure S1). The X chromatids in *S. latifolia* and *S. dioica* are distinguishable only in anaphase A and anaphase B (the second-largest chromosomes, both arms have subtelomeric X43.1 satellite signal), both in males and females (Figure 1 and Figure S2). During telophase, both X chromatids are no longer distinguishable, and all chromatids are equally separated at the opposite poles of the cell (Figure 1 and Figure S2). The gynodioecious species, *S. vulgaris* (a species with no sex chromosomes) was used as an outgroup to *S. latifolia* and *S. dioica*. We found no sister chromatids protruding towards the division plane in *S. vulgaris*, showing that such localization towards the division plane is dependent on sex chromosome size in *S. latifolia* and *S. dioica* (Figure 1).

**Figure 2:**
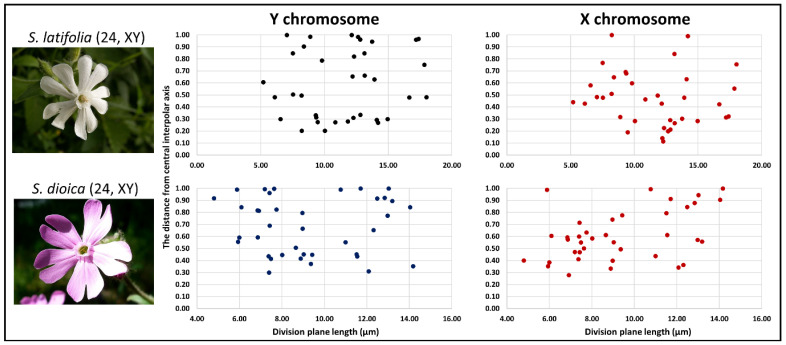
The relative distance of the sex chromosomes from the central interpolar axis. The distance was measured for each studied dioecious plant for both sex chromosomes during anaphase A to telophase. The *X* axis represents the total length of division plane, and the *Y* axis represents the relative distance from central interpolar axis.

Although all autosomal chromatids are moving synchronously with the sex chromosomes during sister chromatid, we tested if the velocity (movement) of the longest chromosome(s) is influenced by its position with respect to the central interpolar axis [34,35]. We measured the distance between the ends of the division planeand the relative distance of both sex chromosomes from the central interpolar axis. We found the Y chromosome located distantly from the central interpolar axis in 54.29% of cells in *S. latifolia* and 68.42% of cells in *S. dioica*. Only a limited number of cells did not possess both large sex chromosomes closer than 10% of the total length of the division cell plane (Figure 2). Further, we found no cells which possessed X and Y chromatids at the central interpolar axis. Compared to the Y chromosome, the X chromosome in both species was distantly located mostly in *S. dioica* (65.79% of cells—more than one-half of observed cells; Supplementary Materials Table S1).

To exclude the impact of hydroxyurea and cell synchronization treatment on sister chromatid division, we performed additional chromosome preparation using the spreading technique from the non-treated apical meristem. We did not find any differences in the sister chromatid division compared to the treated root meristematic cells (DAPI stained, Figure S1). Therefore, the impact of hydroxyurea on the sex chromosome sister chromatid division can be excluded.

**Figure 3:**
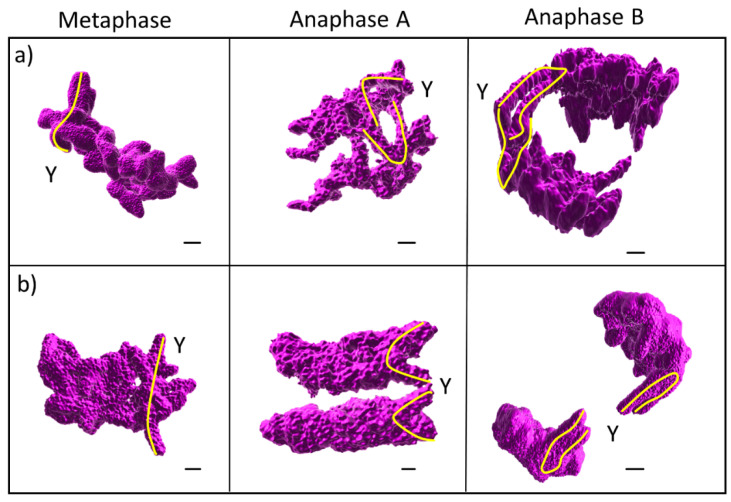
3D confocal analysis during progression from metaphase to Anaphase B. Sister chromatid division in *S. latifolia* (**a**) and *S. dioica* (**b**) male plants using high resolution microscopy. Note the position of the sex chromosomes during transition from metaphase to anaphase B. The longest chromosome was identified based on the length measurement analysis in Imaris software. Scale bar = 2 µm.

To confirm the position of the sex chromosomes during mitotic cell division and to exclude the impact of the chromosome preparation method on their relative distance to the central interpolar spindle axis, we performed 3D high-resolution microscopy on non-synchronised plant material conserved in polyacrylamide pads (Figure 3). In both species, Y chromatids were located distantly to the central spindle axis already in metaphase, following the same positioning during anaphase A and anaphase B, similarly to synchronised cells treated by hydroxyurea (Figure 3A,B). Thus, the position of the sex chromosomes during metaphase and anaphase is conserved among all three types of plant tissue and used technique, surface spreading chromosome preparation from apical meristem, squashing chromosome preparation from the synchronised root meristems, and 3D chromosome conservation using non-synchronised cells in polyacrylamide pads. Nevertheless, it should be stressed that only high-quality chromosome preparations in the case of spreading and squashing techniques were used to score the position of the sex chromosomes during mitosis.

### 2.2. The Spindle Axis Length Is Not Suited for the Large Sex Chromosomes in Gynodioecious Species

**Figure 4:**
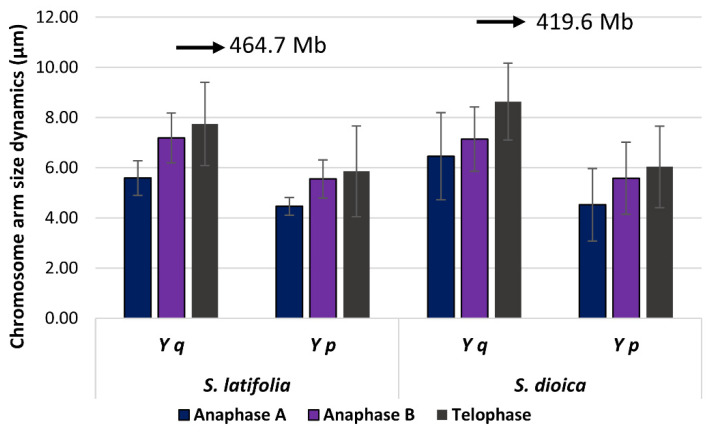
The upper limit of the longest chromosomal arm expansion in *S. dioica* and *S. latifolia*. The expansion limit is calculated from the genome size measurement and the relative chromosome size.

To test the possibility of whether the Y or the X chromosome size interferes with the spindle axis length, we measured its length together with the X and Y chromatid size during the telophase (Tables S2 and S3). Based on the upper limit for the longest arm in the genome [27], the predicted expansion limit of the Y chromosome in *S. latifolia* (with the largest arm 327.3 Mb in telophase) is limited to 464.7 Mb (Table S4) and in males of *S. dioica* to 419.6 Mb (the size of Y q-arm 301.2 Mb) (Table S4). Therefore, the length of the telophase spindle axis is well suited for the large chromosomes and their size in both studied dioecious plants, and further non-recombining region expansion (Figure 4). Nevertheless, in *S. vulgaris*, a plant with a 2.5 times smaller genome compared to the *S. latifolia* and *S. dioica* (Table S5), the length of a spindle axis is 15.37 µm which suggest that the presence of the Y chromosome in the *S. vulgaris* genome will lead to chromosomal break or its elimination (Table S2).

## 3. Discussion

**Figure 5:**
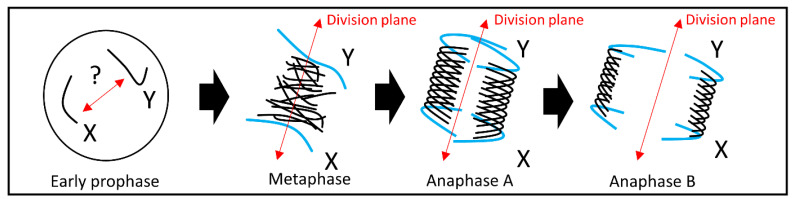
Schematic model showing the position of the large sex chromosomes during mitotic cell division. The distant position of the large chromosomes from central interpolar axis suggests lower chromosome velocity which is higher at the end of the division plane. The X and Y chromosome position in *Silene* suggests chromosome pre-positioning in early prophase. A similar mechanism was proposed for the large chromosomes in *Melanoplus* [35], supported by the mathematical simulations of chromosome stiffness and velocity for the large and small chromosomes, respectively [34].

The overall difference in chromosomal size is not beneficial for the correct development of an organism [30,31]. Based on the observations in *Vicia faba*, individuals with unusually large chromosomes, longer than half of the microtubule spindle axis in telophase, do not divide correctly [27]. The so-called “upper limit of chromosome size”, later confirmed by another study in barley [28], estimates the interference of large chromosomes with mitotic nuclear division. Cell division in such karyotypes which have enlarged chromosomal arms leads to incomplete chromosome separation and formation of micronuclei from the chromosomal breaks during the transition from anaphase to telophase. We demonstrate that the Y chromatids in studied species are still protruding towards the spindle axis and division plane, but we found neither chromosome breaks nor micronuclei. This suggests that the large Y chromosomes non-recombining regions in studied species have not expanded to the point in which they will impede correct cell division (Figure 3). Based on the measurements of the spindle axis length in telophase, both dioecious species are well adapted to the longest chromosome arm (Figure 5, Table S2), yet were found distantly located from the central interpolar axis in the majority of scored cells (Figure 2).

A hypothesis of the chromosome movement during mitosis predicts that the velocity of the chromosome decreases with increasing stiffness and length [34]. As the computational prediction of the sex chromosome stiffness is unknown in *S. latifolia* and *S. dioica*, we assume lower sex chromosome velocity (higher stiffness) compared to the autosomes (based on their length and genomic composition) [12,14,25,36,37]. Nevertheless, the protrusion of the sex chromosomes towards the division plane is only a passive effect of the sex chromosome size. We suggest that the cells posite the sex chromosome distantly from the central interpolar axis during mitosis, compensating the X and Y chromosome size (and stiffness) (Figure 5). This is supported by the distant localization of the largest chromosome in barley [27] and grass hopers [35].

An intriguing question is how the studied dioecious species differ in their cell division progression compared to the gynodioecious *S. vulgaris*, and what is the difference in the division length between sexes. Further, it is not clear how the large sex chromosomes are positioned from the central spindle axis before the cell enters mitosis, e.g., as described for chromosomal domains in *Drosophila* (reviewed in [38]). Based on previous oligo painting DNA probe labelling experiments on interphase nuclei in both studied *Silene* species [15], we assume that the sex chromosomes occupy distant nuclear domains already in early prophase, setting their position during sister chromatid division. Nevertheless, further studies are needed to test such a hypothesis using a more robust set of oligo painting probes, specific to the sex chromosome individual strata.

From the length of the telophase spindle axis [28], we estimate the limit expansion size for Y q-arm chromosome in *S. latifolia* (464.7 Mb) and *S. dioica* (419.6 Mb). The Y chromosomes in *S. latifolia* and *S. dioica* are 1.4 and 1.5–1.6× larger than their X counterparts and this clearly shows that Y chromosome expansion might be limited by the cell division machinery (spindle axis extension), but the expansion might be species specific. The gynodioecious *S. vulgaris* with no sex chromosomes and a 2.5× times smaller genome (Table S5), has a shorter axis length compared to both dioecious species. Although we expected a similar length of the telophase spindle axis, its length explains non-successful hybridization experiments between *S. vulgaris* and *S. latifolia* or *S. dioica*. Further, the shorter spindle axis in telophase agrees with the folding ratio ensuring that the lower length of a chromosome in base pairs generally corresponds to lower chromatin size [31], and mitotic chromosome length scale responses to cell and nuclear size differences [39,40,41].

Although, large heteromorphic sex chromosomes have been described in only 18 plant species to date, similarly as in *S. latifolia* and *S. dioica*, there are several other species that possess large and heteromorphic sex chromosomes, as described in *Coccinia grandis* (the Y is 2.2× larger than the X [14]) or *Rumex acetosa* (the X chromosome is the largest in the genome [12]). The overall Y chromosome size variation between populations of *C. grandis* [42] further shows the ongoing process of non-recombining region expansion which might be limited by its spindle axis length similarly to *Silene*.

## 4. Methods

### 4.1. Material

Seedlings of *S. latifolia*, *S. dioica*, and *S. vulgaris* listed in Table S6 (collection of seeds of Institute of Biophysics of the Czech Academy of Sciences) were used for chromosome preparation using a protocol previously described in [15] with a minor modification. Seeds were washed for 5 s in 50% ethanol, followed by 20 min of surface sterilization in 15% bleach supplemented by 0.1% Tween 80 (Sigma-Aldrich, St. Louis, MO, USA). After 20 min, seeds were shortly washed again in 50% ethanol, in sterile distilled water and kept at 4 °C for one week in the dark. Young seedlings (average size 1 cm) were synchronized for 16 h in 1.125 mM hydroxyurea at RT, 2× washed for 5 min in distilled water, and incubated for 4 h in distilled water at RT again. After 4 h, seedlings were fixed in freshly prepared Clarke’s fixative (ethanol: glacial acetic acid, 3:1, *v*:*v*) for 24 h and used for chromosome preparation.

### 4.2. DNA Isolation and Probe Preparation

Fresh tissue material was obtained from plants growing in a greenhouse kept in long-term daylight term conditions (16 h light/8 h dark). DNA was isolated according to the manufacturer’s instructions using the Plant Genomic DNA Kit (Sigma-Aldrich, St. Louis, MO, USA, G2N70). STAR-C and X43.1 were amplified according to [15]. After amplification, PCR products were purified by QuiaQuick PCR Purification Kit (Qiagen, Hilden, Germany, 28104), checked on 1% agarose gel and labelled by a standard nick translation kit according to the manufacturer’s instructions using Atto488 NT (PP-305L-488), Atto550 NT (PP-305L-550) and Cy5 (PP-305L-647N) (Jena Bioscience, Jena, Germany). All probes were purified following the manufacturer’s instructions. Chromosomes were counterstained with DAPI in VectaShield antifade solution (Vector, Burlingame, CA, USA, H-1500).

### 4.3. Chromosome Preparation and Image Measurements

Chromosomes were prepared as described in [15] with minor modifications. Fixed root tips were digested for 40–80 min in a 1% enzyme mix diluted in 0.001 M citrate buffer. Squashed chromosomes (on slides) were incubated for 5 min in freshly prepared Clarke’s fixative and used directly for fluorescence in situ hybridization (FISH) or stored at −20 °C in 96% ethanol until use. For the surface spreading chromosome preparation method from the apical meristem, a protocol was followed as described in [43] with the same enzyme mix used for root digestion.

Six slides for females, males, and one gynodioecious species with the highest number of anaphase A, anaphase B, and telophase spreads were screened for structurally conserved, non-damaged cells without any chromosomal or microtubule distortion (reviewed in [44]). The slides and nuclei were used for FISH experiments, division plane, and chromosomal arm length measurements (30–40 nuclei measured per species). FISH was performed as described by [15]. In *Silene* species, STAR-C hybridises to primary constriction and two additional regions on the Y [45]. X43.1 hybridises to the subtelomeric region, depicting the PAR on the Y and X chromosomes [37]. Chromosome images were captured as described in [15]. Flower images (Figure 2) downloaded on: https://commons.wikimedia.org/wiki/Main_Page, accessed on 13 December 2021.

### 4.4. 3D Microscopy Analysis

Chromosomes for 3D analysis measurements were prepared following the protocol of [46], using polyacrylamide pads. Confocal laser scanning microscopy was performed on inverted microscope Zeiss Axio Observer7 with laser scanning unit LSM880 equipped with four solid-state lasers (405 used), Plan-Apochromat 63x/1.40 oil objective, 32 channel spectral GaAsP detector, and Airyscan detector (increased resolution and signal to noise ratio). Images were captured using ZEN Black software, and 3D images were generated from Z-stack with fixed slice width and analysed using Imaris software (v. 9.8, Bitplane, Oxford Instruments, Abingdon-on-Thames, UK).

## Supplementary Materials

The following material are available online at www.mdpi.com/article/10.3390/ijms23052422/s1.
