# Supplementary material for: The Sister Chromatid Division of the Heteromorphic Sex Chromosomes in Silene Species and Their Transmissibility towards the Mitosis
Source: Int J Mol Sci. 2022 Feb 22;23(5):2422. doi: 10.3390/ijms23052422 (PMC8910698; doi:10.3390/ijms23052422)
Supplement: Supplementary file 1 [file ijms-23-02422-s001.zip › Figure S1.pdf]

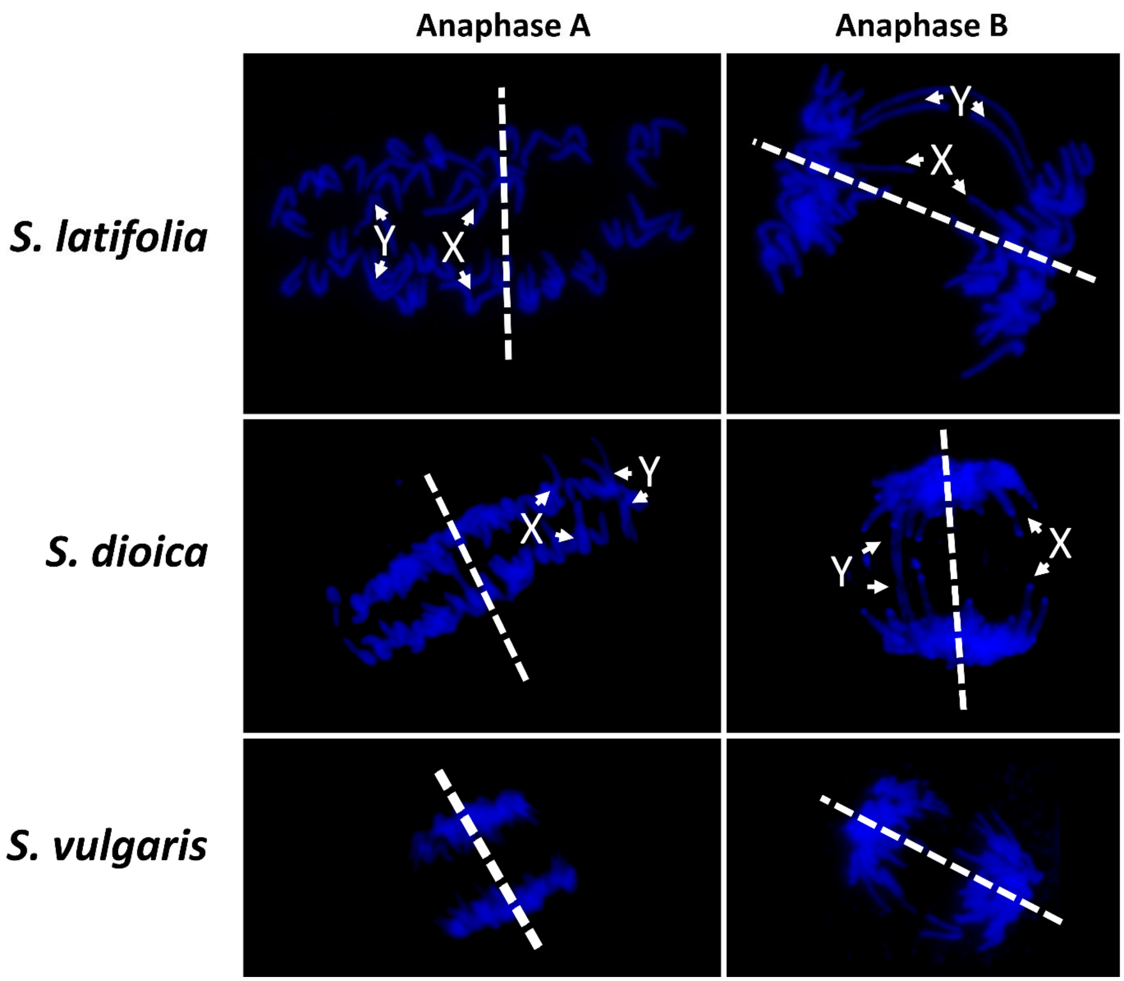


**Figure S1** – Cell division in apical meristem without chemical treatment in *S. latifolia, S. dioica* and *S. vulgaris.* Note the position of the sex chromosomes (marked by arrows) and their distance from the central interpolar axis (dash lines). Scale bar = 10 μm
