# Supplementary material for: The Sister Chromatid Division of the Heteromorphic Sex Chromosomes in Silene Species and Their Transmissibility towards the Mitosis
Source: Int J Mol Sci. 2022 Feb 22;23(5):2422. doi: 10.3390/ijms23052422 (PMC8910698; doi:10.3390/ijms23052422)
Supplement: Supplementary file 1 [file ijms-23-02422-s001.zip › Figure S2.pdf]

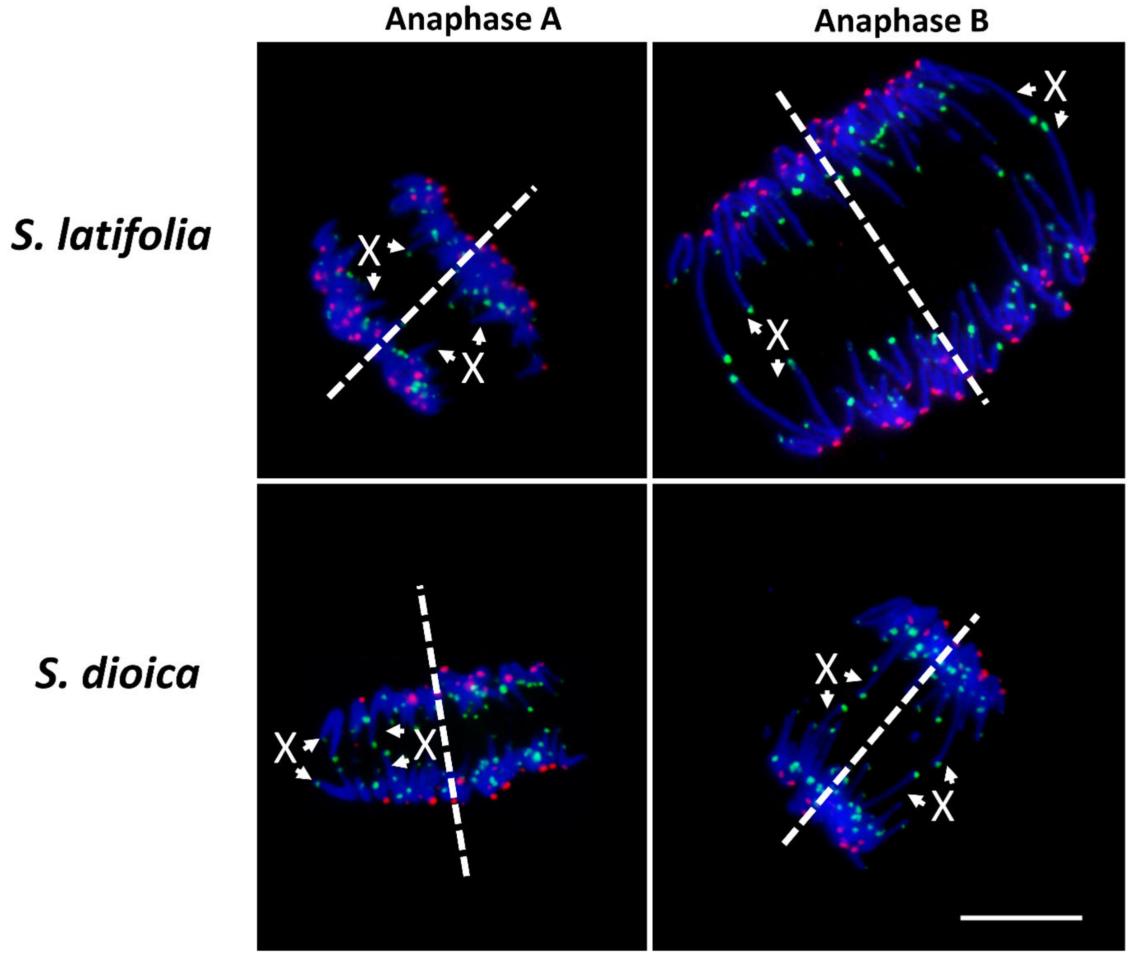

Figure S2 – Mitotic cell division in *S. latifolia* and *S. dioica* female individuals. Subtelomeric satellite X43.1 (green) probe was used as a marker for Y q-arm chromosomes and STAR-C (red) as a marker for primary constriction in *S. latifolia*, *S. dioica* and *S. vulgaris*. Chromosomes were counterstained with DAPI (blue). Note the position of the sex chromosomes (marked by arrows) and their distance from the central interpolar axis (dash lines). Scale bar = 10 μm
