# Supplementary material for: The Sister Chromatid Division of the Heteromorphic Sex Chromosomes in Silene Species and Their Transmissibility towards the Mitosis
Source: Int J Mol Sci. 2022 Feb 22;23(5):2422. doi: 10.3390/ijms23052422 (PMC8910698; doi:10.3390/ijms23052422)
Supplement: Supplementary file 1 [file ijms-23-02422-s001.zip › Table S1-S6.pdf]

Table S1 – The position of the largest chromosome in anaphase-telophase transition

	chromatids at the edge		chromatids close to central interpolar axis	
**Species**	Y (%)	X (%)	Y (%)	X (%)
S. latifolia	54.29	34.29	45.71	65.71
S. dioica	68.42	65.79	31.58	34.21

Table S2 – The length of the spindle axis during anaphase A, anaphase B and telophase in studied species

Species	anaphase A (µm)	+/-	anaphase B (µm)	+/-	Telophase (µm)	+/-
S. latifolia	7.39	0.59	15.39	2.74	21.98	1.71
S. dioica	7.29	1.88	15.33	2.11	24.03	2.07
S. vulgaris	7.66	0.82	11.24	0.84	15.37	1.01

Table S3 – The X and Y chromosome arm length during anaphase A, anaphase B and telophase in studied species

Species		anaphase A (µm)	+/-	anaphase B (µm)	+/-	Telophase (µm)	+/-
S. latfiolia	Y q	5.59	0.69	7.19	1.00	7.74	1.66
	Y p	4.46	0.35	5.55	0.76	5.86	1.81
S. dioica	Y q	6.46	1.74	7.14	1.28	8.63	1.53
	Y p	4.52	1.44	5.58	1.44	6.04	1.62

Table S4 – The comparison of chromosomal arm length and predicted arm limit size for the longest arm in studied dioecious plants during telophase

Species	Length of longest arm (µm/Mb) [a]	+/-	Length of the spindle axis in telophase (µm)[b]	+/-	Predicted upper limit for the longest arm extension (µm/Mb)[c]
S. latifolia	7.74 (327.3 Mb)	1.66	21.98	1.71	10.99 (464.7 Mb)
S. dioica	8.63 (301.2 Mb)	1.53	24.03	2.07	12.02 (419.6 Mb)

[a] the length estimated from the average longest arm chromosomal length in telophase

[b] the length estimated from the most two distal points (centromere) in telophase

[c] the length estimated = the length of the spindle axis in telophase/2

*the length of the X chromosome

Table S5 – The genome and the sex chromosome size according to their chromosomal length

Species	Genome size (Mb) [b]	2n	Y (%)[c]	+/-	Y (Mb)	+/-	X (%)[c]	+/-	X (Mb)	+/-
S. latifolia [a]	**5535.5**	24, XY	10.4	1.9	575.1	106.8	7.2	1.1	397.9	62.8
S. dioica [a]	**5398.6**	24, XY	9.5	1.4	512.0	77.4	6.4	0.4	346.7	20.3

[a] – the size of the *S. vulgaris* genome = 2210.3 Mb

[b] – genome size estimated from 2C (pg) Kew mean value (*978Mb)

[c] – relative size (%) of the sex chromosome estimated from the average sex chromosome length in c-metaphase (µm)/whole genome length (µm), 10 cells counted

Table S6 – Plant material

Species	Population	No. of chromosomes	The biggest chromosome	Origin
Silene latifolia	U16*	24, XY	Y	Bačovský et al. 2020**
Silene dioica	Tišnov	24, XY	Y	
Silene vulgaris	Čertovica (CERT)	24		

*population made by 16 generations of full-sib mating

** Bačovský V, Čegan R, Šimoníková D, et al (2020) The Formation of Sex Chromosomes in Silene latifolia and S. dioica Was Accompanied by Multiple Chromosomal Rearrangements . Front. Plant Sci. 11:205
